# Successful conversion therapy of advanced gallbladder carcinoma by chemotherapy combined with immunotherapy: two case reports

**DOI:** 10.3389/fonc.2026.1758176

**Published:** 2026-03-05

**Authors:** Long Li, Bing Tong, Wei Gong, Haisheng Xu, Xiaofeng Liao, Huapeng Sun

**Affiliations:** 1 School of Medicine, Wuhan University of Science and Technology, Wuhan, China; 2 Department of General Surgery, Xiangyang Central Hospital, Affiliated Hospital of Hubei University of Arts and Science, Xiangyang, China; 3 Department of Oncology, Xiangyang Central Hospital, Affiliated Hospital of Hubei University of Arts and Science, Xiangyang, China

**Keywords:** chemotherapy, conversion therapy, gallbladder cancer, immunotherapy combined therapy, radical operation

## Abstract

Gallbladder cancer (GBC) is a highly aggressive malignancy of the biliary system and is frequently diagnosed at an advanced stage upon initial presentation. Nonetheless, the introduction of conversion therapy has allowed certain initially inoperable cases of GBC to become candidates for radical resection after comprehensive modalities, including chemotherapy, immunotherapy, and radiotherapy. In the current retrospective analysis, we present two patients with initially unresectable GBC treated at Xiangyang Central Hospital, affiliated with Hubei University of Arts and Science. After receiving a combination of chemotherapy and immunotherapy, substantial tumor regression was observed at both primary and metastatic sites, achieving partial remission according to the Response Evaluation Criteria in Solid Tumors (RECIST). Conversion therapy proved effective, enabling both patients to undergo successful radical resections. Following surgery, the original therapeutic regimen was continued until completion of five cycles. Follow-up extended through November 2025, with no evidence of tumor recurrence observed.

## Introduction

Gallbladder cancer (GBC) ranks sixth in incidence among gastrointestinal malignancies and represents the most prevalent subtype of biliary tract cancer (BTC). This disease exhibits aggressive biological behavior, subtle initial clinical symptoms, a low rate of surgical resection, and a high postoperative recurrence rate, collectively contributing to extremely poor patient prognoses. Currently, radical surgical resection remains the sole potentially curative intervention for GBC (1–4). However, due to its insidious onset and diagnostic challenges, over half of GBC patients in China are diagnosed at advanced stages, thereby precluding the option of curative surgery at initial presentation (5).

Palliative chemotherapy is commonly employed as the primary therapeutic approach for advanced GBC that is unsuitable for resection. However, its therapeutic effectiveness remains significantly inferior to radical surgical intervention, resulting in unsatisfactory patient survival outcomes. Therefore, identifying methods to facilitate surgical resection in advanced GBC cases constitutes a pivotal strategy for enhancing long-term prognosis. Recently, conversion therapy has emerged as a promising approach, potentially transforming tumors previously classified as unresectable into lesions amenable to surgical removal, thus broadening the possibility of curative treatment.

At present, a standardized clinical approach for conversion therapy in BTC remains unavailable. Since the ABC-02 trial in 2010, gemcitabine plus cisplatin (GC) has become the accepted first-line chemotherapy regimen for patients with advanced BTC (6). Nevertheless, the therapeutic outcomes of GC have been consistently limited, with minimal progress noted over the following decade. Recently, two landmark clinical studies, TOPAZ-1 and KEYNOTE-966, demonstrated that integrating PD-1/PD-L1 inhibitors into standard gemcitabine-cisplatin chemotherapy markedly enhanced clinical efficacy in advanced BTC patients (7–9). Consequently, combined treatment strategies incorporating PD-1/PD-L1 immune checkpoint inhibitors and chemotherapy have become an active area of investigation for advanced BTC management.

Conversion therapy for GBC has not yet been widely implemented. Currently, clinical practice largely relies on protocols developed for BTC, with limited adaptation specific to GBC. Furthermore, existing studies are predominantly composed of individual case reports, lacking robust support from large-scale clinical research. In light of this evidence gap, this paper presents two cases of advanced GBC in which patients were successfully converted to resectability and underwent radical surgery following a novel combination regimen of chemotherapy and immunotherapy, aiming to provide valuable clinical insights for the development of conversion therapy strategies in advanced GBC.

## Case description

**Figure 1:**
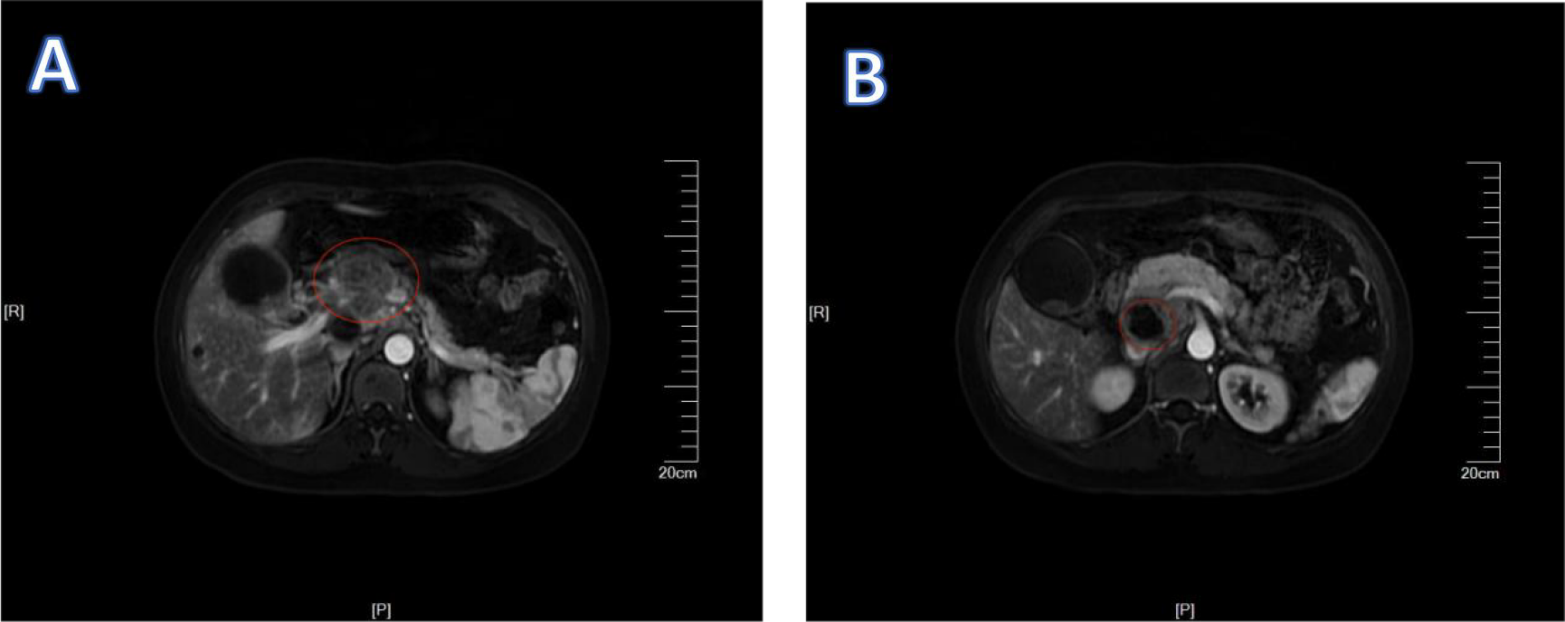
1Patient 1’s MRI results. The red circles in **(A, B)** indicate swollen lymph nodes.

**Figure 2:**
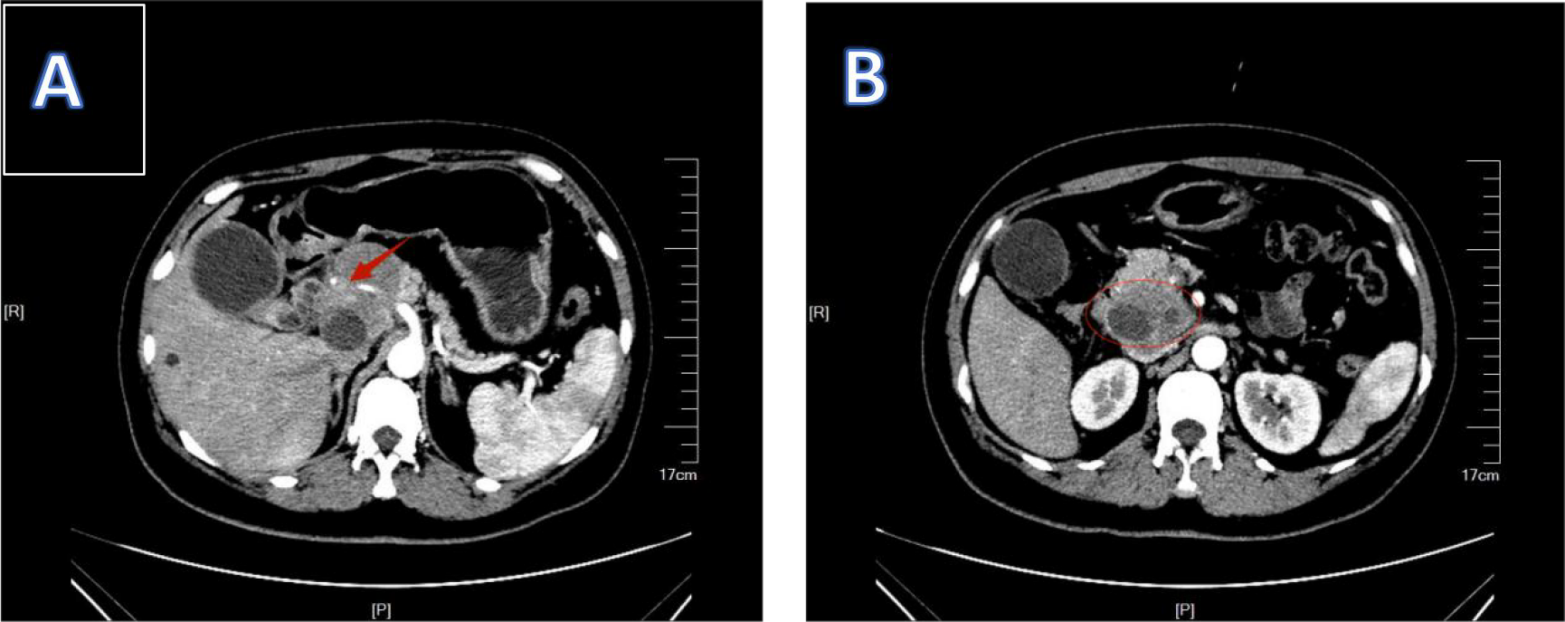
2Patient 1’s enhanced CT results. **(A)** shows that the lymph nodes are surrounding the hepatic artery. **(B)** shows enlarged lymph nodes.

**Figure 3:**
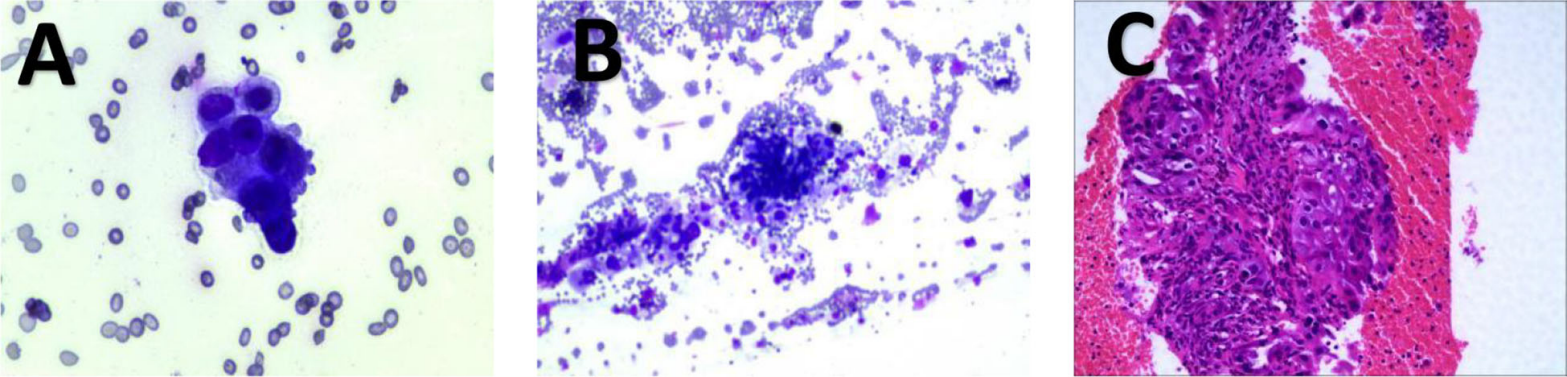
1Patient 1’s pathological biopsy results. **(A)** shows the smear of the puncture material from the hepatic hilar lymph nodes. **(B)** shows the puncture and irrigation fluid from the lymph nodes in the hepatic hilum. **(C)** shows the lymph nodes at the hepatic hilum.

Patient 1, a 45-year-old female, presented to the Department of Hepatobiliary Surgery at Xiangyang Central Hospital on January 4, 2024, reporting upper abdominal discomfort persisting for one week. General physical examination did not reveal any clinically significant abnormalities or positive findings. Elevated tumor marker levels were noted: CEA 100.44 ng/mL, CA-125 59.03 U/mL, CA-153 28.31 U/mL, and CA-199 38.98 U/mL; AFP remained within normal limits. MRI findings suggested gallbladder carcinoma accompanied by hepatic hilar lymph node enlargement (**Figure 1**). Enhanced CT imaging demonstrated metastatic lymphadenopathy at hepatic portal and portocaval regions (**Figure 2**). Ultrasound-guided percutaneous biopsy was performed on abdominal lymph nodes, and cytological evaluation confirmed poorly differentiated adenocarcinom (**Figure 3**). Immunohistochemical (IHC) analysis revealed positivity for CK18, CK19, CK7, and Claudin4, while the hepatocyte marker was negative. The Ki-67 proliferation index was 30%. CEA (polyclonal) expression was negative, whereas P53 showed weak to strong positivity, with a positivity rate of 60%.

**Figure 4:**
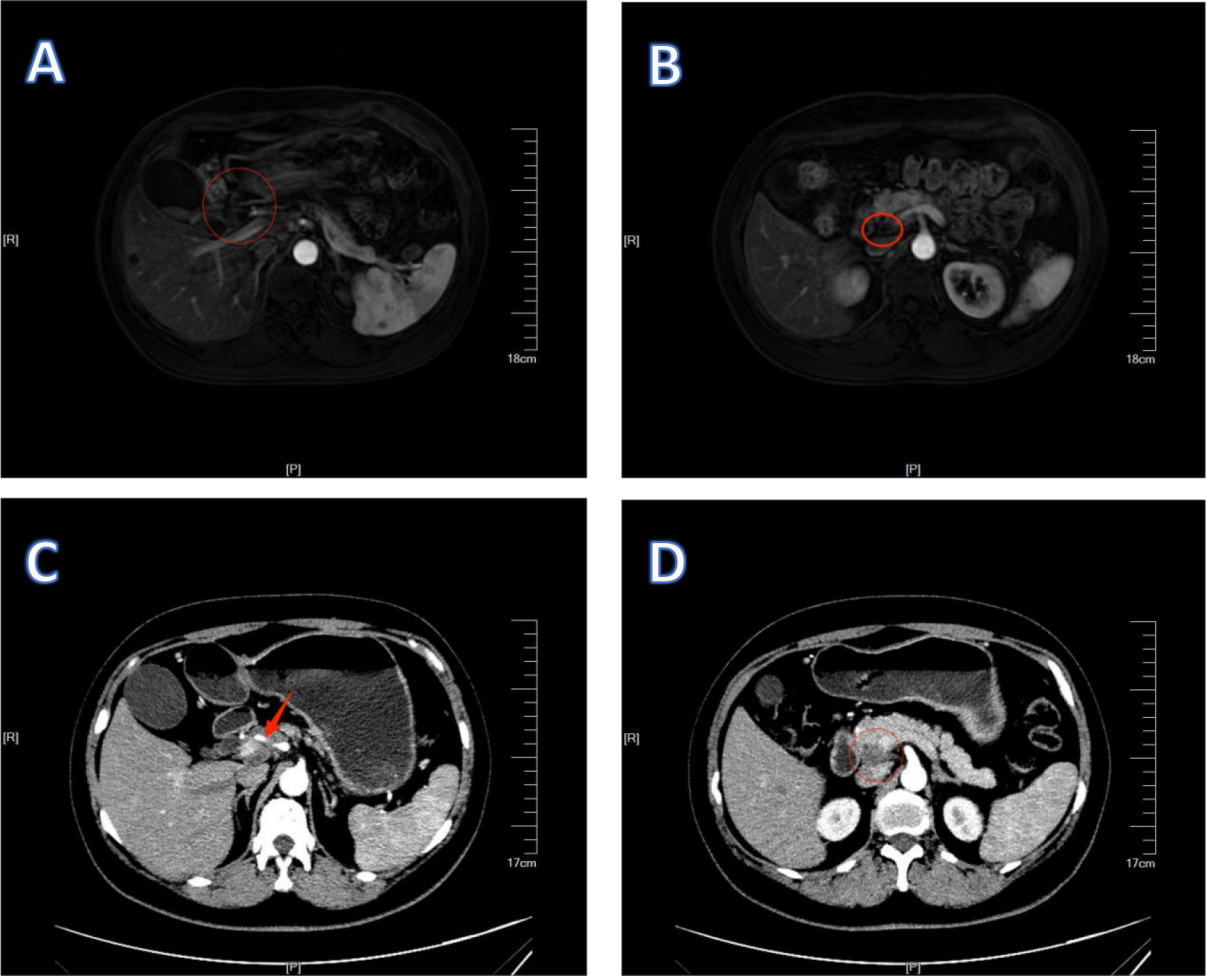
1Preoperative examination of patient 1. **(A, B)** are MRI images, showing that the lymph nodes have shrunk. **(C, D)** are CT images. **(C)** shows an improvement in the condition where the lymph nodes surround the hepatic artery.

Following a comprehensive assessment by the multidisciplinary team (MDT) and according to the 8th edition of the American Joint Committee on Cancer (AJCC) TNM staging system for GBC, the patient was classified as stage IIIB (T3N2M0). Conversion therapy is recommended as the initial treatment strategy. The patient was fully informed about potential risks associated with treatment, and conversion therapy commenced on January 6, 2024. The treatment protocol administered to patients included combined chemotherapy and immunotherapy: camrelizumab (200 mg on day 2), nab-paclitaxel (200 mg on days 1 and 8), and S-1 (50 mg, administered twice daily from day 1 to day 14), with a planned total of five cycles. After completing two cycles, one patient exhibited myelosuppression, characterized by a reduced absolute neutrophil count of 0.85 × 10^9^/L. According to the National Cancer Institute’s Common Terminology Criteria for Adverse Events (CTCAE v5.0), this adverse reaction was categorized as grade 3 neutropenia. Following administration of granulocyte colony-stimulating factor (G-CSF), laboratory parameters normalized. Following completion of three cycles, contrast-enhanced CT and MRI scans demonstrated reduction of the gallbladder carcinoma lesions, along with significant regression of multiple lymph nodes in the hepatic hilum and portocaval space compared to earlier imaging (**Figure 4**). Re-examination of tumor markers showed a CEA level reduced to 8.48 ng/mL, while AFP, CA-125, CA15-3, and CA19–9 returned to normal ranges. According to the RECIST, the overall response was assessed as partial response (PR), indicating treatment efficacy.

**Figure 5:**
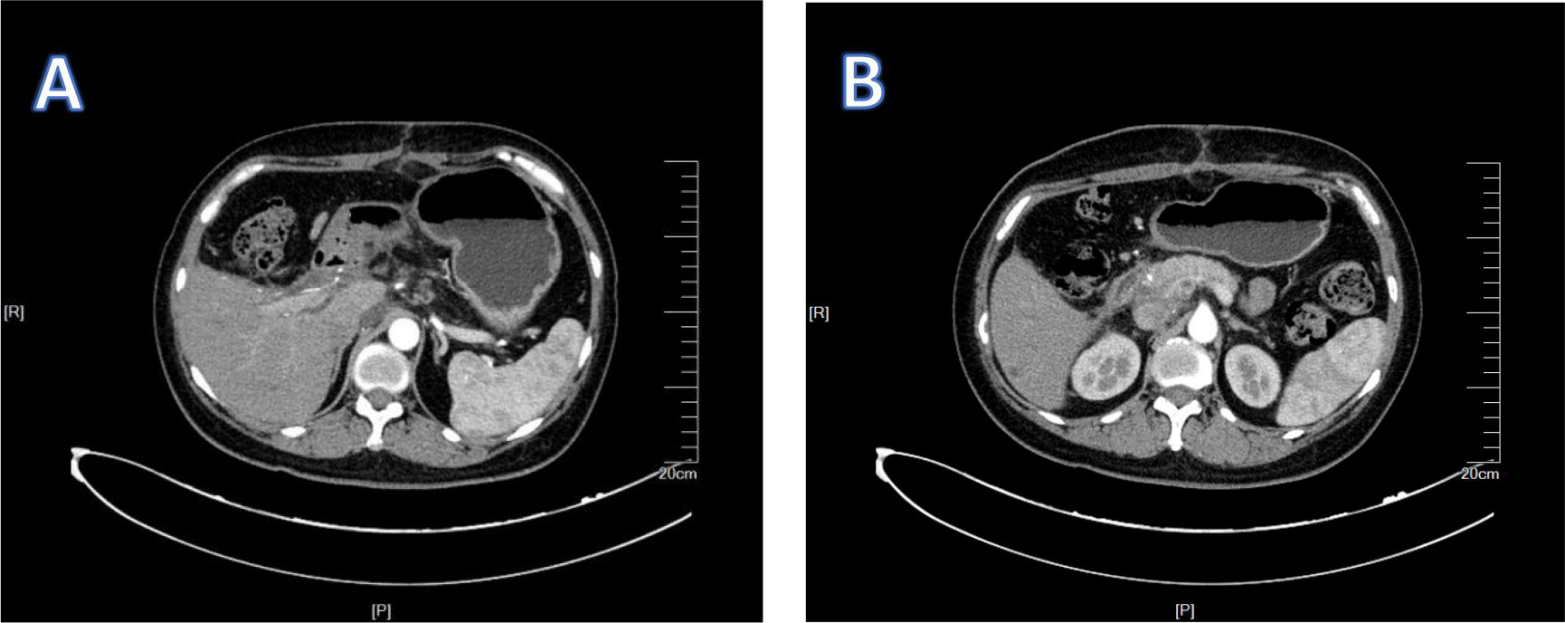
1Postoperative examination of patient 1. **(A, B)** show the CT examination results of patient 1 after the operation.

**Figure 6:**
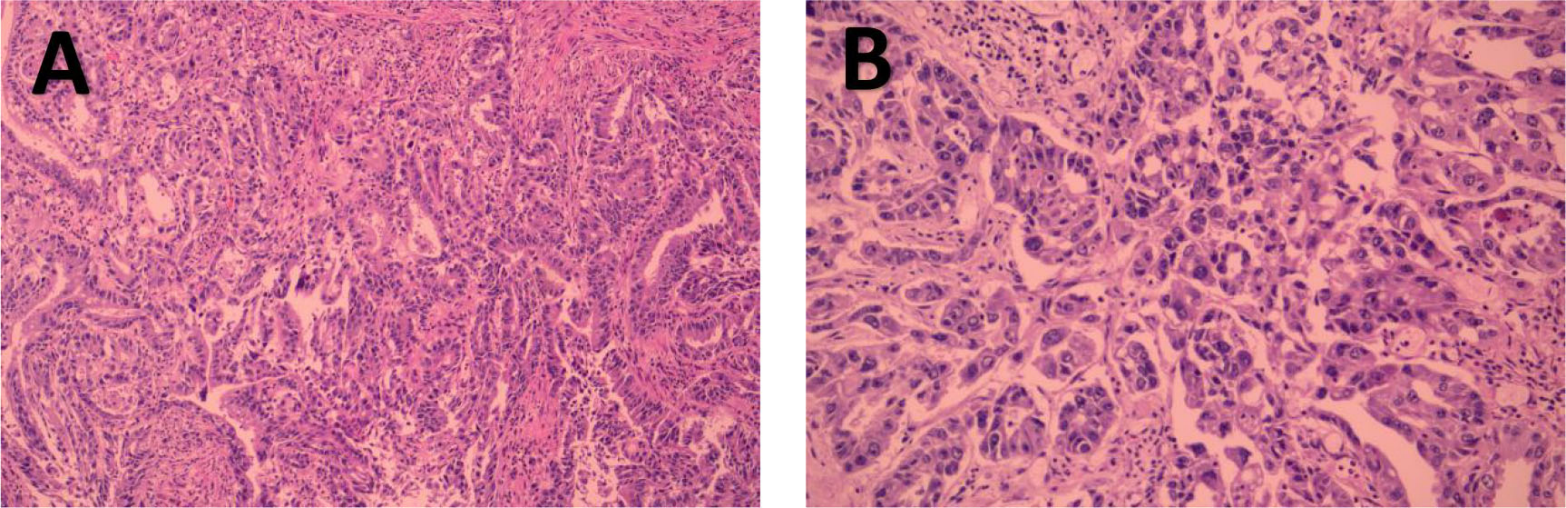
2Postoperative pathological examination results of patient 1. **(A, B)** show the microscopic pathological images of part of the liver and gallbladder of patient 1 after the operation.

**Figure 7:**
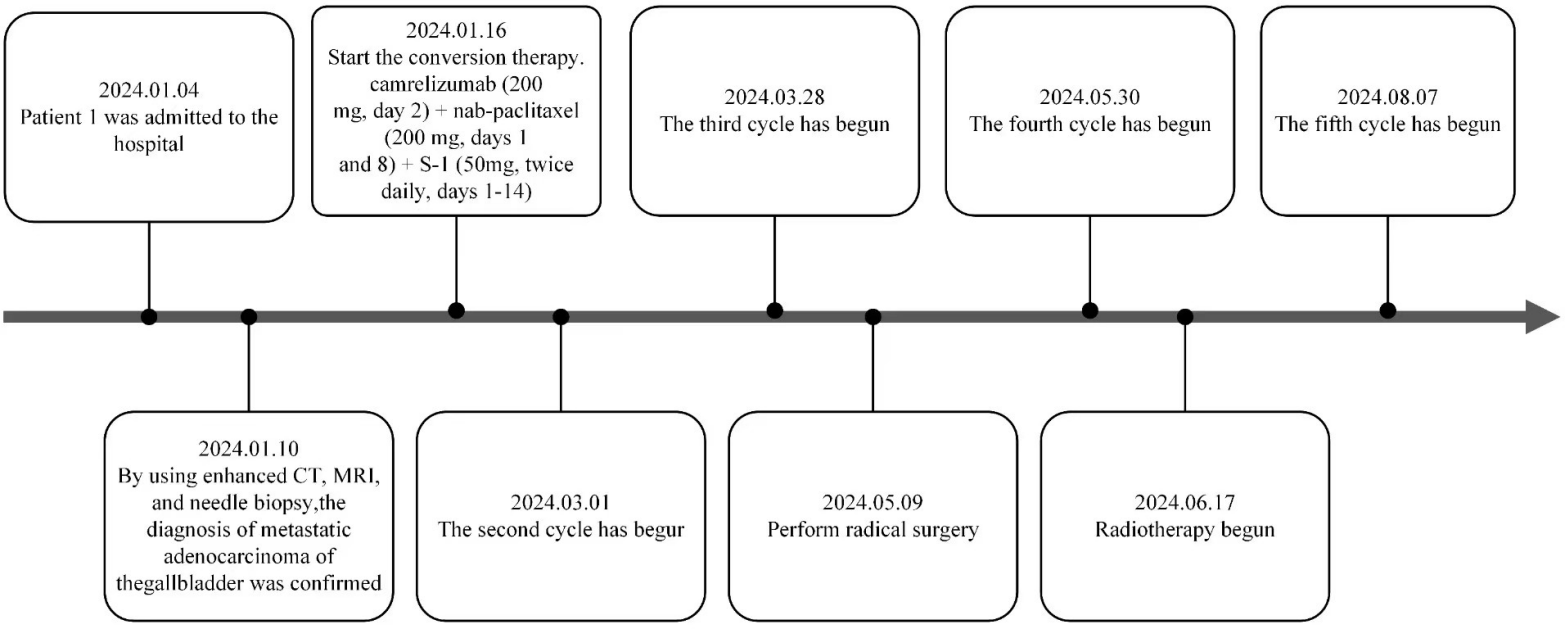
1Treatment timeline of patient 1.

On May 9, 2024, the patient underwent laparoscopic exploration followed by open hepatic segmentectomy (S4b + S5), cholecystectomy, and regional lymphadenectomy (**Figure 5**). Postoperative pathology confirmed invasive adenocarcinoma of the gallbladder body (**Figure 6**). The patient recovered and was discharged postoperatively, subsequently continuing treatment with camrelizumab plus albumin-bound paclitaxel and S-1 for another two cycles (**Figure 7**). Radiotherapy was initiated following the fourth cycle (PTV: 1.8 Gy × 10 fractions; PTV: 1.8 Gy × 15 fractions). Regular postoperative follow-ups continued through November 2025, covering a duration of 17 months, during which CT scans revealed no evidence of recurrence or lymph node metastasis.

**Figure 8:**
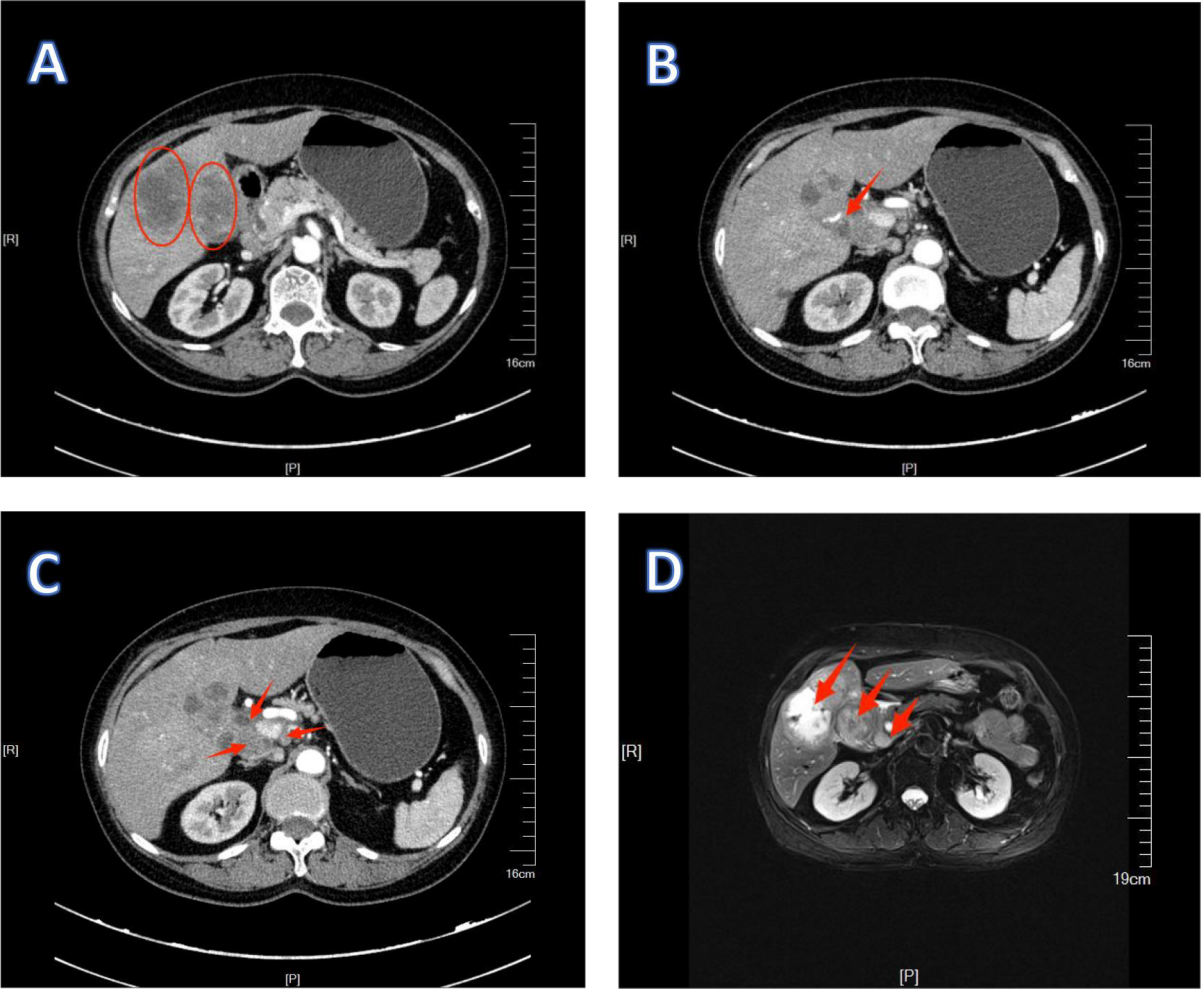
1Imaging findings of GBC in patient 2 without any treatment. **(A–C)** are CT images. **(D)** is an MRI image. **(A)** shows the primary lesion of gallbladder cancer and the infiltrating lesion in the right lobe of the liver. **(B)** shows that the lymph nodes surround the hepatic artery. **(C)** shows lymph node metastasis around the hepatic hilum.

**Figure 9:**
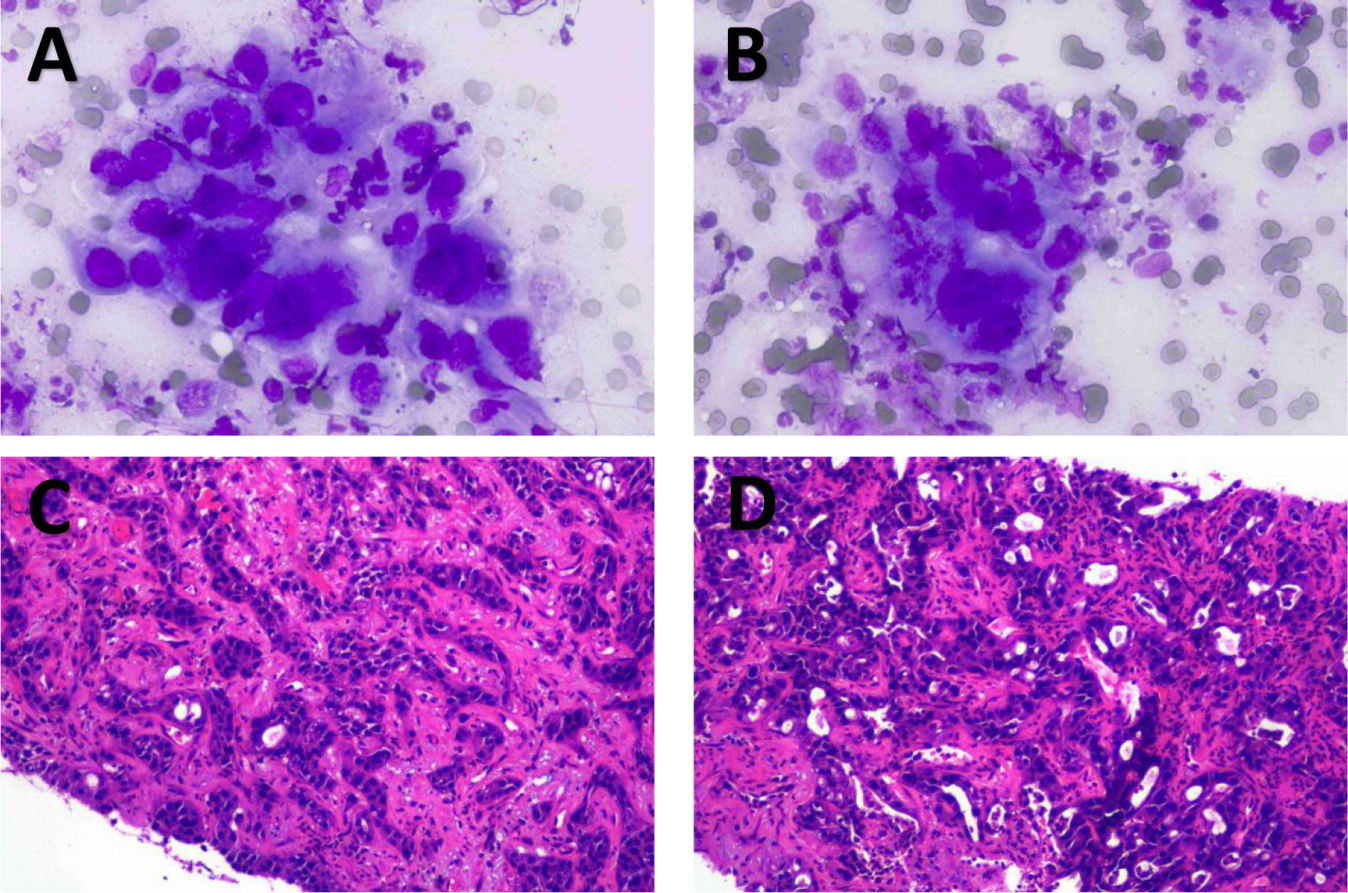
1Pathological biopsy results of patient 2. **(A, B)** show the results of the liver biopsy smear from patient 2; **(C, D)** show the microscopic findings of the liver lesion in patient 2.

Patient 2, a female, presented at the Oncology Department of Xiangyang Central Hospital on October 28, 2024, with a chief complaint of “hepatic space-occupying lesion identified over one week prior.” Tumor marker analysis showed elevated levels of CEA (7.32 ng/mL) and CA125 (120.00 U/mL), while CA72-4, CA19-9, and AFP remained within normal limits. Enhanced MRI imaging revealed gallbladder carcinoma accompanied by multiple intrahepatic and hepatic hilar lymph node metastases. Additionally, enhanced CT scans demonstrated infiltration of gallbladder carcinoma into the right hepatic lobe, along with extensive metastatic involvement of hepatic hilar lymph nodes (**Figure 8**). CT-guided biopsy of hepatic lesions indicated poorly differentiated carcinoma accompanied by inflammatory reactions (**Figure 9**). Immunohistochemical staining yielded positive results for CK7, CK18, and CK19, but negative outcomes for CK20, hepatocyte antigen, and Glypican-3. p53 protein expression was heterogeneously positive, consistent with a wild-type phenotype, while Ki-67 expression reached 40%. Immunohistochemistry also confirmed positive staining for mismatch repair proteins MSH-1, MSH-2, MSH-6, and PMS-2.

**Figure 10:**
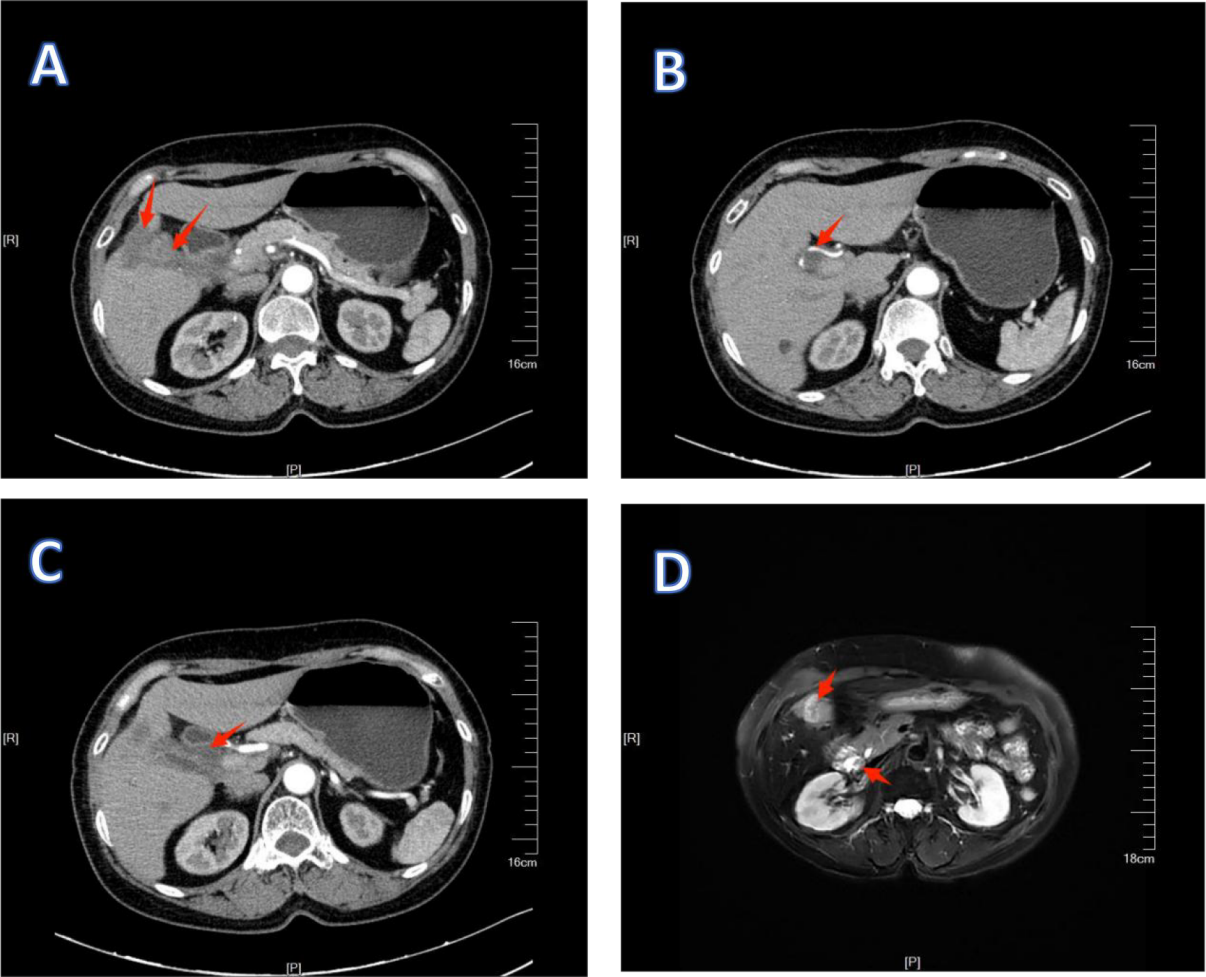
1Preoperative examination of patient 2. **(A–C)** are CT images. **(D)** is an MRI image. **(A)** shows the reduction of the primary lesion and the metastatic lesion. **(B)** shows an improvement in the condition where the lymph nodes surround the hepatic artery. **(C)** shows a reduction and shrinkage of lymph node metastasis around the hepatic hilum.

Based on the comprehensive analysis of clinical manifestations, imaging findings, and pathological results, and in accordance with the NCCN Clinical Practice Guidelines in Oncology: Biliary Tract Cancers (2024.V5), the patient was diagnosed with GBC staged as T3N2M0. After a thorough multidisciplinary team (MDT) assessment, conversion therapy was recommended as the initial treatment. The patient was fully informed about potential risks associated with treatment, and conversion therapy commenced on January 6, 2024. The patient underwent the same therapeutic regimen comprising camrelizumab (200 mg on day 2), nab-paclitaxel (200 mg on days 1 and 8), and S-1 (50 mg, twice daily, days 1–14), also scheduled for five cycles. No grade 3 or 4 adverse reactions related to treatment were observed during the entire treatment duration. After four treatment cycles, contrast-enhanced CT and MRI scans demonstrated a reduction in gallbladder carcinoma lesions and a decrease in both the size and number of metastatic lymph nodes in the hepatic hilar region (**Figure 10**). Re-examination of tumor markers showed a CEA level of 8.45 ng/mL, with AFP, CA125, CA19-9, and CA72–4 all within normal reference ranges.

**Figure 11:**
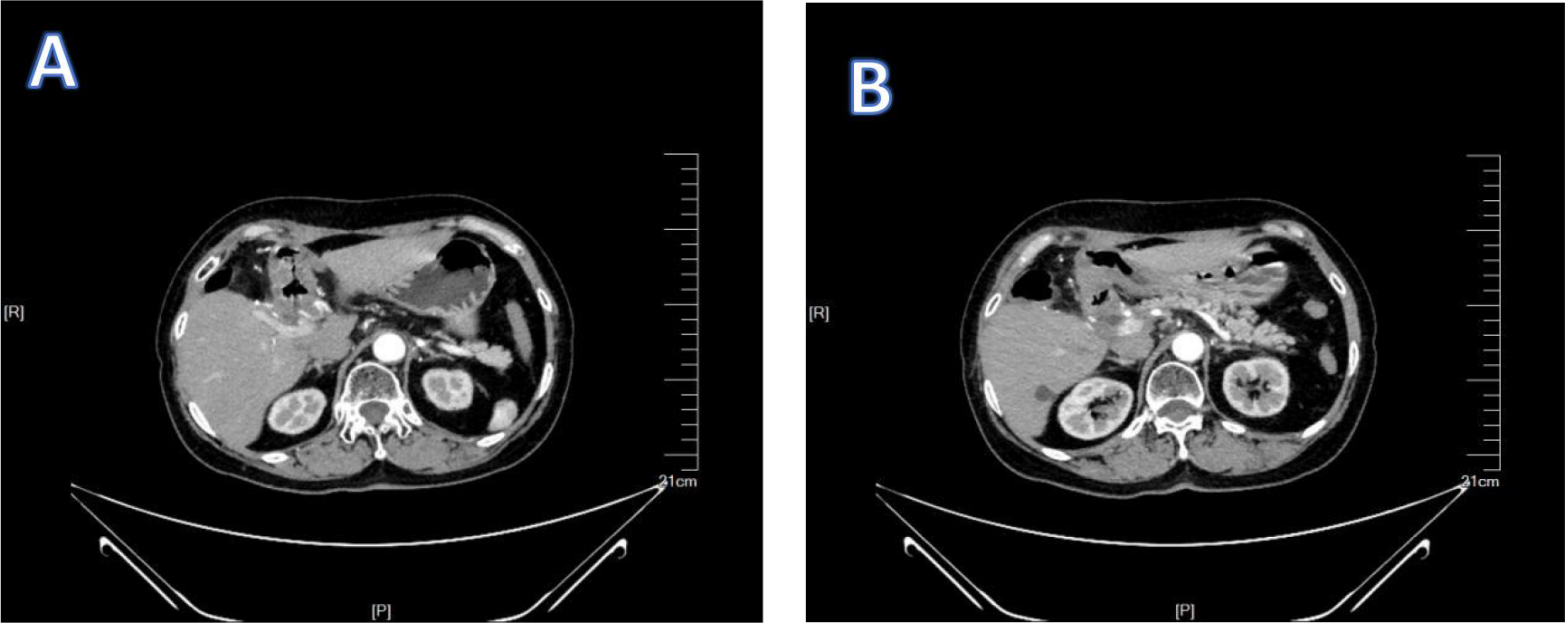
1Postoperative examination of patient 2. **(A, B)** show the CT examination results of patient 2 after the operation.

**Figure 12:**
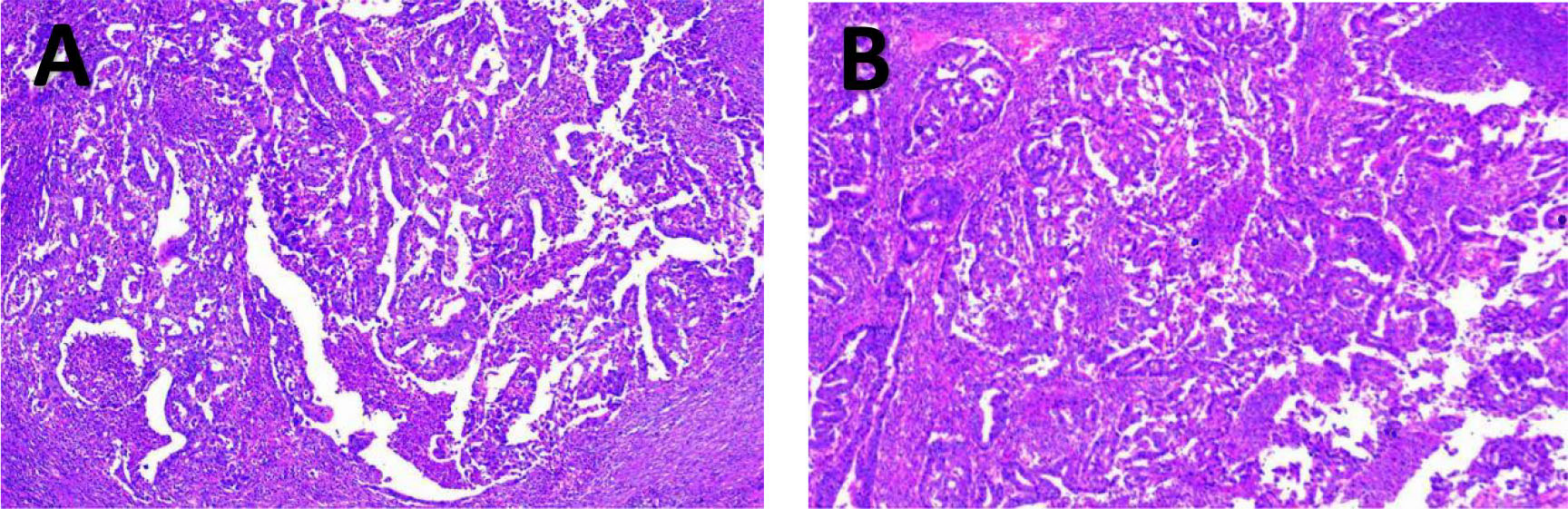
2Postoperative pathological examination results of patient 2. **(A, B)** show the microscopic pathological images of part of the liver and gallbladder of patient 2 after the operation.

**Figure 13:**
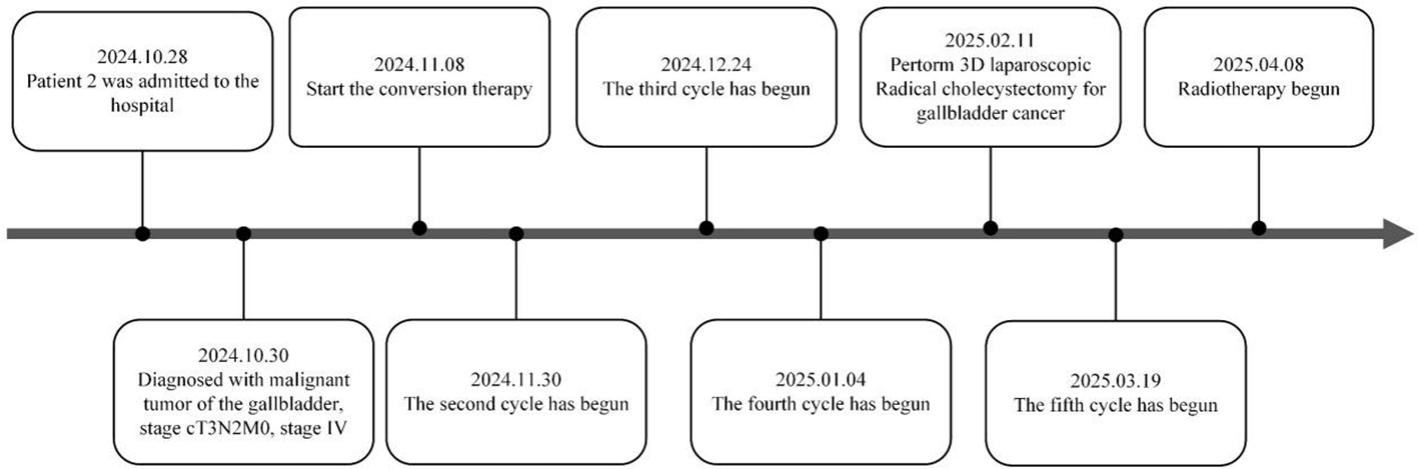
1Treatment timeline of patient 2.

On February 11, 2025, the patient underwent 3D laparoscopic radical resection of gallbladder carcinoma, involving resection of hepatic segments S4b + S5 + partial S6, cholecystectomy, regional lymph node dissection, and excision of omental lesions (**Figure 11**). Postoperative pathological examination revealed moderately to poorly differentiated gallbladder adenocarcinoma with extensive necrosis (**Figure 12**). Microscopic examination of intrahepatic carcinoma tissue showed widespread necrotic changes accompanied by fibrosis of the surrounding liver tissue, inflammatory cell infiltration, and histiocytic proliferation, consistent with post-chemotherapeutic alterations. No lymph node metastasis was observed. According to the RECIST criteria, the overall response was assessed as a complete response (CR). The patient recovered uneventfully and was discharged postoperatively, continuing treatment with the remaining conversion therapy cycle (**Figure 13**). Regular postoperative follow-up continued, and as of November 2025, after over eight months, no recurrence or metastasis had been observed.

## Discussion

Gallbladder carcinoma (GBC) is a highly malignant neoplasm of the digestive system, typically diagnosed at advanced stages, resulting in limited opportunities for curative surgical intervention. The recent development of conversion therapy has introduced novel therapeutic possibilities for these patients. Conversion therapy, widely adopted in gastrointestinal malignancies such as colorectal cancer, gastric cancer, and hepatocellular carcinoma, principally aims to achieve tumor downstaging via systemic pharmacological treatment, thus facilitating surgical resection with curative intent (10–12). Clinical evidence increasingly suggests the feasibility and efficacy of conversion therapy in patients with biliary tract cancer (BTC), significantly improving the likelihood of radical surgical resection.

The ABC-02 study identified gemcitabine combined with cisplatin (GC) as the first-line chemotherapy standard for advanced BTC, reporting a median overall survival (OS) of around 11.7 months among treated patients (6). However, subsequent studies have demonstrated limited efficacy of the GC regimen when used as conversion therapy, along with relatively severe toxicities that result in suboptimal tolerance in certain patients (13, 14). In response to these limitations, a phase III randomized controlled trial (JCOG 1113) in Japan was conducted to evaluate the therapeutic efficacy of gemcitabine plus S-1 (GS regimen) in patients with advanced or recurrent BTC. The results showed that, compared with the standard GC regimen, the GS regimen significantly prolonged median OS (15.1 months *vs*. 13.4 months). In subgroup analyses involving patients with locally advanced GBC, the advantages of the GS regimen became even more evident. Median OS improved substantially from 13.9 months (GC regimen) to 20.2 months (GS regimen), and median progression-free survival (PFS) also increased (6.8 months versus 5.8 months). Moreover, the GS regimen showed a reduced frequency of grade 3 or 4 adverse events, including anemia, thrombocytopenia, and peripheral neuropathy, demonstrating better patient tolerability (13). A study on a modified GS regimen (TG 1380) reported satisfactory therapeutic efficacy and favorable safety (14); however, its overall effectiveness remains insufficient to meet current clinical demands.

In recent studies, the combination regimen of gemcitabine, cisplatin, and albumin-bound paclitaxel (GAP) has been reported to notably enhance OS outcomes in advanced BTC patients (15–17). Nonetheless, the clinical efficacy of this regimen remains controversial. Certain investigations indicate that the GAP regimen, compared to the standard GC protocol, does not significantly improve either OS or PFS. Specifically, the phase III randomized controlled trial SWOG S1815 found no significant OS advantage of GAP over GC, with a higher occurrence of adverse events in the GAP arm. However, subgroup analyses revealed greater improvements in OS and PFS in GBC patients compared with other BTC subtypes when treated with the GAP regimen (18).

In recent years, immune checkpoint inhibitors (ICIs), particularly antibodies targeting programmed cell death receptor-1 (PD-1) and its ligand PD-L1, have shown remarkable clinical efficacy across multiple cancer types. Studies exploring genomic alterations involving ERBB2/ERBB3 in GBC suggest that these genetic mutations contribute to tumor immune evasion via upregulation of PD-L1. The PD-1/PD-L1 interaction suppresses cytotoxic T-cell responses, allowing GBC tumor cells to escape host immune detection (19). This mechanism provides a strong theoretical foundation supporting the use of ICIs in GBC treatment.

The TOPAZ-1 study, a randomized, double-blind, placebo-controlled, international phase III trial, enrolled 685 treatment-naive advanced BTC patients randomly assigned to receive either the PD-L1 inhibitor durvalumab combined with GC chemotherapy or placebo plus GC (7, 8). Interim analysis demonstrated significantly prolonged median OS in the durvalumab arm compared to controls (12.8 months *vs*. 11.5 months; hazard ratio [HR] 0.80, 95% confidence interval [CI] 0.66–0.97; P = 0.021), as well as improved median PFS (7.2 months *vs*. 5.7 months; HR 0.75, 95% CI 0.63–0.89; P = 0.001). These outcomes strongly support durvalumab plus GC as an effective first-line therapeutic approach in advanced BTC.

Another phase III randomized controlled trial, the KEYNOTE-966 trial, randomly assigned 1,069 patients with BTC to receive either pembrolizumab plus GC or placebo plus GC (9). The median OS was 12.7 months in the pembrolizumab group versus 10.9 months in the control group (hazard ratio [HR] 0.83, 95% confidence interval [CI] 0.72–0.95), and the median PFS was 6.5 months versus 5.6 months, respectively (HR 0.87, 95% CI 0.76–0.99). Both of these landmark studies confirm that combining PD-1/PD-L1 inhibitors with gemcitabine-cisplatin chemotherapy provides substantial clinical benefit for patients with advanced BTC and supports this combination as a recommended first-line treatment option. Additionally, a single-arm phase II trial investigated camrelizumab combined with gemcitabine plus oxaliplatin (GEMOX) for advanced BTC, reporting an objective response rate (ORR) of 54% and a median OS of 11.8 months (20, 21). These results highlight the therapeutic promise of integrating ICIs with chemotherapy as first-line treatment for advanced BTC, including GBC

Since 2019, Xiangyang Central Hospital, affiliated with Hubei University of Arts and Science, has actively explored conversion therapy strategies for advanced BTC, employing a combination regimen comprising camrelizumab, albumin-bound paclitaxel, and S-1. The current study was ethically approved by the Medical Ethics Committee of Xiangyang Central Hospital (Approval No.: 2022-083-005). The triple-agent therapy produces potent antitumor effects via synergistic mechanisms: Camrelizumab, a PD-1 inhibitor, binds specifically to PD-1 on T-cell surfaces, thereby disrupting PD-1/PD-L1 signaling, counteracting tumor-mediated immune evasion, and enhancing T-cell-mediated cytotoxic responses against tumor cells (22, 23). Albumin-bound paclitaxel exerts direct cytotoxic effects by disrupting microtubule formation, inhibiting tumor cell division, promoting apoptosis, facilitating the release of tumor-associated antigens, and enhancing CD8+ T-cell proliferation and differentiation, thereby remodeling the tumor immune microenvironment (24–26). Additionally, S-1 is an oral fluoropyrimidine prodrug that undergoes preferential conversion into the active metabolite 5-fluorouracil (5-FU) within tumor cells. It suppresses tumor proliferation by interfering with DNA and RNA synthesis, complementing the antimicrotubule activity of albumin-bound paclitaxel and targeting a broader range of cell cycle phases. Moreover, previous studies have demonstrated that 5-FU can upregulate PD-L1 expression in various tumor tissues (27–30). Therefore, this study hypothesizes that 5-FU may similarly induce PD-L1 expression in GBC tissues, thereby increasing tumor immunogenicity. In summary, the combination of these three agents enables multidimensional synergy involving immune modulation, direct tumor cell killing, and sustained inhibition of tumor proliferation, ultimately enhancing overall antitumor efficacy.

This treatment regimen has demonstrated promising clinical efficacy in relevant studies involving patients with advanced intrahepatic cholangiocarcinoma and hilar cholangiocarcinoma (31). Building on the successful experience with conversion therapy for these two subtypes of BTC at our institution, two patients with advanced GBC were selected for conversion therapy using this triple-agent regimen following a MDT discussion. After completion of the predefined treatment protocol, both patients exhibited significant tumor regression, reduced regional lymph node metastases, and downstaging from unresectable locally advanced disease to resectable status. Both subsequently underwent radical surgical resection. Postoperative pathological examination revealed extensive tumor necrosis, confirming achievement of the intended conversion effect. During follow-up, Patient 1 has been monitored for 17 months post-surgery without evidence of recurrence or lymph node metastasis. Patient 2 has completed over 8 months of follow-up, and both patients remain free of disease during the observation period. Follow-up is ongoing.

The combination therapy regimen employed in this study offers several advantages. First, it demonstrates a significant therapeutic effect. The synergy between chemotherapy and immunotherapy enables a more comprehensive antitumor response, allowing for direct tumor cell killing as well as activation of the host immune system, thereby reducing the risk of tumor recurrence and metastasis. Second, the regimen exhibits favorable safety. During treatment, apart from Patient 1 who developed granulocytopenia, all other adverse reactions were primarily gastrointestinal in nature and mild in severity. No grade 3 or 4 adverse events were observed in the remaining patients.

However, the current study has several limitations. Firstly, biomarkers that may influence immunotherapy efficacy, including PD-L1 expression status, tumor mutation burden (TMB), and microsatellite instability (MSI), were not examined; thus, associations between these biomarkers and therapeutic response could not be assessed. Secondly, this case report involves only two patients, significantly limiting the generalizability of the findings due to the absence of large-scale clinical data. Consequently, further studies with expanded patient cohorts are needed to confirm the efficacy and reproducibility of the treatment regimen for the broader GBC patient population.

In summary, this study employed conversion therapy utilizing a triple-agent regimen of camrelizumab, albumin-bound paclitaxel, and S-1 in patients diagnosed with initially unresectable advanced GBC. The therapy effectively transformed previously inoperable lesions into surgically resectable tumors, enabling successful radical resections and favorable clinical outcomes for both patients. These preliminary results suggest that this combination regimen is both feasible and efficacious for conversion therapy in advanced GBC, warranting further exploration as a novel treatment strategy for this challenging patient population.

## Data Availability

The original contributions presented in the study are included in the article/supplementary material. Further inquiries can be directed to the corresponding authors.
